# Topological Traps Control Flow on Real Networks: The Case of Coordination Failures

**DOI:** 10.1371/journal.pone.0015210

**Published:** 2010-12-09

**Authors:** Carlos P. Roca, Sergi Lozano, Alex Arenas, Angel Sánchez

**Affiliations:** 1 Chair of Sociology, in particular of Modeling and Simulation, Department of Humanities, Social and Political Sciences, ETH Zurich, Zurich, Switzerland; 2 Grupo Interdisciplinar de Sistemas Complejos (GISC), Departamento de Matemáticas, Universidad Carlos III de Madrid, Leganés, Spain; 3 Departament d'Enginyeria Informàtica i Matemàtiques, Universitat Rovira i Virgili, Tarragona, Spain; 4 Instituto de Biocomputación y Física de Sistemas Complejos (BIFI), Universidad de Zaragoza, Zaragoza, Spain; 5 Instituto de Ciencias Matemáticas CSIC-UAM-UC3M-UCM, Madrid, Spain; Indiana University, United States of America

## Abstract

We study evolutionary games in real social networks, with a focus on coordination games. We find that populations fail to coordinate in the same behavior for a wide range of parameters, a novel phenomenon not observed in most artificial model networks. We show that this result arises from the relevance of correlations beyond the first neighborhood, in particular from topological traps formed by links between nodes of different degrees in regions with few or no redundant paths. This specificity of real networks has not been modeled so far with synthetic networks. We thus conclude that model networks must be improved to include these mesoscopic structures, in order to successfully address issues such as the emergence of cooperation in real societies. We finally show that topological traps are a very generic phenomenon that may arise in very many different networks and fields, such as opinion models, spread of diseases or ecological networks.

## Introduction

Understanding interactions among people and their social contacts is a key issue for the comprehension of the manners in which society works and how everybody's welfare can be improved. This problem is related with, but not limited to, the emergence of cooperation in human and animal societies [1], [2] as well as in other contexts (e.g., the formation of multicellular organisms or their organs [3]). A mathematical tool that has led to many deep insights about interactions among individuals is game theory, particularly in evolutionary form [4]–[6], as it allows to formulate in quantitative terms the most important prototypical social interactions, such as conflicts and/or dilemmas [7]. To apply this tool to understand social human behavior, the proper setting is to specify what is the network of relationships [8]–[10] among the intervening agents. We thus arrive at evolutionary game theory on graphs, one of the most intriguing dynamical processes on networks and one that is currently receiving a lot of attention [11], [12]. In this context, a great deal of research has considered the Prisoner's Dilemma [13] on artificially designed model networks as the paradigm to understand the emergence of cooperation, and a plethora of results have been obtained concerning the positive or negative influence of networks in sustaining cooperative strategies (see [11], [12] for recent reviews). Subsequent research on wider classes of games has provided a more accurate view of the intricate relationship between population structure as given by model networks, strategy update dynamics and type of dilemma [14]–[17]. However, real social networks may have features (such as, e.g., hierarchical levels of organization or communities) that are not well captured by model networks and that may have an important effect on the emergence and evolution of cooperation. Thus, in spite of its original aim, evolutionary game theory on graphs has not addressed the problem of cooperation in human societies in a sufficiently realistic manner yet.

In view of this, among the necessary ingredients for a realistic model of social interaction, we here focus on improving the description of the underlying interaction network. To that end we use real social networks as the substrate on which the model games will be played, our aim being to scrutinize the incidence of the topology on the evolutionary outcome. It is important to note that studies restricted to the Prisoner's Dilemma [18], [19] have already reported that different social networks with apparently similar characteristics can lead to largely different behavior. To gain a deeper insight on the effect of the network, we enlarge the range of possible social interactions to consider. As we will see, this allows us to pinpoint coordination games such as the Stag Hunt (see below) as the proper microscope to probe the details of the network. Thus, we show the emergence of coordination failures and uncover the mechanism behind it: topological traps connecting different groups of nodes in regions where redundant paths are scarce, which turn out to be responsible for the failure of global coordination in the network. It is important to note, however, that our findings are relevant much beyond the realm of the social sciences. As we will argue in the concluding discussion, topological traps may be present in many real networks that exhibit different types of slowing down or even stopping of dynamical phenomena, in a very similar manner to the one we have found with coordination games.

## Results

### Games on social networks

As a realistic approach to modeling social behavior, we have used two social substrates obtained by sampling real relational data. We have chosen these substrates instead of other social network data available, such as the IMDB network for actor collaboration in movies or scientific collaboration networks, because their links are defined through true personal exchanges. In contrast, in those other data links are defined by joining the collaboration framework (movies, research projects, articles, etc.), which does not necessarily imply mutual interaction. Our first substrate is a social network obtained from the email traffic between members of the University Rovira i Virgili (in Tarragona, Spain), where nodes represent individual email addresses and undirected links between two nodes indicate bidirectional communication (at least one email in each direction) [20]. Our second real social substrate consists of nodes representing users of the “Pretty-Good-Privacy” encryption algorithm, where links trace trust relationships between those persons who sign each other's public keys [21]. For a comparison of some of their statistical properties see [18].

Regarding the interaction among agents, we consider a wide set of 2×2 social dilemmas. They consist in games with 2 players who choose between 2 strategies and with no difference in role. While by no means this is the most general scenario of interaction between individuals, understanding binary interactions is a first and crucial step towards dealing with more complex, *n*-ary settings. Games are defined by the following payoff matrix
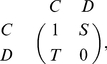
(1)where rows represent the strategy of the player who obtains the payoff and columns that of her opponent, player and opponent being any of the two individuals. Strategies are labeled C and D for cooperate and defect, because we interpret the game as a social dilemma. Restricting the values of the coefficients within the intervals −1<*S*<1 and 0<*T*<2, we have the Harmony game (HG from now on) [22] (0<*S*, *T*<1) and three classic social dilemmas: Prisoner's Dilemma (PD) (−1<*S*<0,1<*T*<2), Stag Hunt (SH) [23] (−1<*S*<0<*T*<1), and Snowdrift (SD) [24] (also called Hawk-Dove [25]) (0<*S*<1<*T*<2). Each game corresponds, thus, to a unit square in the *ST*-plane, which in turn corresponds to a specific tension context in the social interaction under consideration [26]. Indeed, the Prisoner's Dilemma, the reference game in much of the published work on this issue, poses a very demanding scenario on cooperation, subject to both the temptation to defect and the risk in cooperation [26]. The Stag Hunt game, however, is less problematic in the sense that it is a coordination game dominated by the risk in cooperation alone, or in other words, by the question of trust. Note that in PD the conflict is between individual rationality and mutual benefit, whereas in SH one player's rational choice depends on her beliefs about what the other will do. In addition, there are situations that apparently correspond to a PD which are in fact better modeled by means of a SH. This is the case, for instance, when the PD is repeated and the players have in mind the “shadow of the future” [27], i.e., the possibility of future interactions, or for psychological reasons [28], or else when group selection is taken into account [29]. In addition, we also include the Snowdrift game, which isolates the other tension present in PD, namely the temptation to defect, avoiding the undesirable consequences of being defected upon, because in SD mutual defection becomes the worst case scenario.

We have carried out simulations of the evolution of populations playing these games and embedded in our two real social networks, PGP and URV. See Methods for a detailed description of the simulations.

### Cooperation in 2×2 games

**Figure 1:**
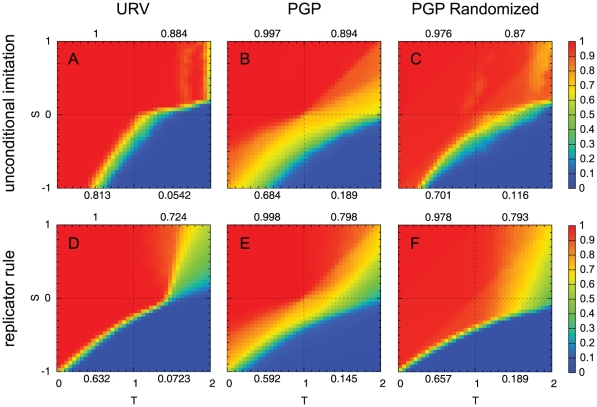
Cooperation maps for different values of *S* and *T* show coordination failures on the PGP network. Asymptotic density of cooperators for 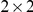 social dilemmas on the URV (left), PGP (middle) and degree-preserving randomized PGP networks (right). Top row: Unconditional imitation rule; bottom row: replicator rule. In the upper left quadrant of each panel we have the Harmony game (0<*S*, *T*<1), in the upper right quadrant the Snowdrift game (0<*S<1<T<2*), in the bottom left the Stag Hunt game (−1<*S<0<T<1*), and in the bottom right the Prisoner Dilemma (−1<*S<0*, *1<T<2*). Numbers above and below each quadrant represent the average value of the cooperation level in the quadrant. Note the anomalous smooth transition in the PGP network (middle column), which indicates the existence of coordination failures. For comparison, the URV network shows a very small region where coordination is not achieved, comparable to the results of model networks. The randomized version (not shown) gives essentially the same results as the original network, indicating the absence of peculiar features in its topology.

In Fig. 1 we present the results of the asymptotic (or stationary) cooperation density obtained from the simulations in the PGP network, the URV email network and a randomized version of the PGP network. The general appearance of the results resembles those found in highly heterogeneous or scale-free networks [14], with a large increase of the cooperation level in the SD quadrant (note that the numbers appearing in the plots over or below each quadrant represent the average value of the cooperation level in that quadrant). The most salient feature, however, arises from the comparison of results on the PGP network with those on the URV network: the PGP network shows a smooth transition in the SH quadrant (bottom left), from cooperation to defection, which to the best of our knowledge has never been reported with model networks [11], [12]. This transition is observed both under the replicator rule and the unconditional imitation rule, which implies that this is not a consequence of the presence or lack of stochasticity in the dynamics. We have also verified that this result is asymptotic, in the sense that longer simulation times do not change it, and that it does not result from an average over realizations with asymptotic homogeneous states (all cooperators or all defectors), but all realizations with the same parameter set end up with very similar heterogeneous outcomes. This is certainly an striking result, as it means that there is a wide range of game payoffs in which the population ends up in a mixed-strategy or polymorphic state, without converging to one of the two Nash (also evolutionary) equilibria of the basic game. Therefore, this is a true characteristic of the evolutionary outcome of coordination games on the PGP network, which makes it different from both mean-field or well-mixed population results and from all model networks studied so far, and whose origin we have to unveil. Furthermore, as Fig. 1 shows, this effect disappears when the original network is randomized, by rewiring it while preserving the degree of each node, following a procedure introduced in [30]. Notice also that the anomalous heterogeneous states appear around the sharp transition where the population is expected to behave in a bistable manner, so that the effect mostly takes place in the region of SH and part of PD games. Finally, the fact that the URV network shows only a small region of coordination failures, and that its randomized version does not give rise to significant differences with the original one, suggests that the phenomenon we are reporting on the PGP network is deeply engraved in the details of the topology, in so far as their global characteristics are similar.

### Coordination failures

**Figure 2:**
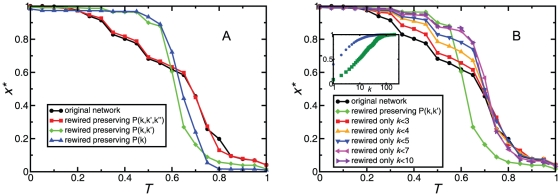
Effect of correlation-preserving rewirings on the evolutionary outcome, along the parameter diagonal *S* = −*T* (SH quadrant). Panel A: Stationary density of cooperators as a function of *T*, for substrates constructed by rewiring the PGP network but preserving degree correlations up to the indicated order. We observe that the smooth transition, which characterizes the coordination failure in the PGP network, disappears when degree correlations of order 3 are destroyed, indicating that the origin of this phenomenon is related to such correlations. Panel B: Influence of bottom-up rewirings. Black and green symbols correspond, respectively, to the original PGP network and a randomized version preserving degree correlations up to order 2. Symbols in other colors correspond to intermediate cases, where only nodes with the indicated degrees are rewired. We observe that the change between the extreme cases (sharp vs. smooth transitions) depends on the degree of the nodes involved in the rewiring process. Inset of Panel B: normalized cumulative distributions of number of nodes (blue circles) and number of stubs (green squares) vs node degree *k*, of the PGP network. Nodes of lower degrees are the majority, but their stubs represent a much smaller portion in the total network.

This initial observation leads us to concentrate our study on the explanation of the continuous transition observed in the SH quadrant with the PGP network, when coordination failures appear and the differences with the asymptotic behavior observed on model networks are dramatic. We focus on the diagonal *S = *-*T*, and study the evolutionary outcome with selective rewirings of the original network, which allow us to activate or deactivate correlations for high or low degree nodes (see Methods). The results of this analysis are reported in Figure 2. It shows that the selective deactivation of correlations does alter the essence of the transition. To begin with, rewirings that preserve not only the degree distribution, but also the degree-degree correlations, make the smooth transition disappear almost completely, reducing the range where coordination failures are observed to a very narrow one around *T = 0.6*. On the contrary, if the rewiring preserves the three-node correlations, the result is practically the same as on the original PGP network, even though the number of rewirings is still significant. To shed further light on this phenomenon, we resorted to randomizations in which only nodes with degree smaller than or equal to a given one were affected by the rewirings. In this case, Figure 2 shows that these rewirings mostly affect the *T<0.6* region, bringing more and more nodes into cooperation. Importantly, the plot also shows that it is enough to work with nodes of degree 7 or less (see purple line on right panel of Fig. 2) to suppress most of the coordination failure region. Admittedly, these nodes represent a large portion of the network (more than 80%, see inset of Fig. 2B). However, the quantity to assess the amount of rewiring performed is the distribution of stubs, i.e. of half links that emerge from each node. The inset of Fig. 2B shows that the stubs of nodes with degree 7 or less account for less than 50% of the total amount.

### Topological traps

**Figure 3:**
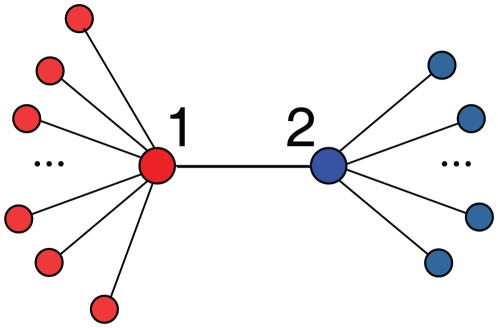
Topological traps are obstacles to the growth of clusters of equal strategists. A simple example is a link between two nodes of different degrees, connected to all cooperators (in red) or all defectors (in blue). See main text for a discussion.

To understand and explain the above results, it is important to recall the evolution of coordination games on model networks. As it was shown in [16], the evolution of coordination games on degree homogeneous networks (e.g., lattices or random homogeneous networks) proceeds in two stages: an initial one in which isolated cooperators disappear whereas regions with high local density of cooperators engulf their defector neighbors and form compact clusters, and a subsequent stage in which these clusters grow and end up making the whole population coordinate in the cooperative equilibrium. This second stage takes place for an appropriate parameter region (along the restriction line *S* = -*T*, it ranges around the interval 0.5<*T*<0.7, depending on the specific network of choice). Therefore, there must be a mechanism preventing this two-step process to take place in the PGP network. We believe that such mechanism arises from the combination of two main features: degree heterogeneity and scarcity of redundant paths, which altogether give rise to topological traps for the propagation of the cooperative strategy. As a simple example, let us consider the configuration displayed in Fig. 3, with a link between two nodes of different degrees k_1_>k_2_. Let us assume for the moment that node 1 is a cooperator with all the neighbors except node 2 being cooperators, and node 2 is a defector fully surrounded by defectors except for node 1. For the two dynamics we are considering here, the strategy of a given node changes (under unconditional imitation) or can change (under the replicator rule) only if it has a neighbor with larger payoff. In this case, node 2 becoming a cooperator requires k_1_>*T*−*S*+1. If we consider now the opposite case, when node 2 is a cooperator and node 1 is a defector, then cooperation spreads when k_2_>*T*−*S*+1. Therefore, we see that if k_1_>k_2_, i.e. when there is degree heterogeneity, for some range of game parameters cooperation can spread from node 1 to node 2, but not vice versa. This implies that “coordination waves” will not propagate over the network uniformly and, when there are no redundant paths available, they will not reach certain regions of the network. On the other hand, for games such that the evolution of the population does not feature these coordination waves, the effect of topological traps is not expected to take place. Accordingly, the heterogeneous states which are found with the PGP network, and which are lost when the network is randomized (see Fig. 1), mostly occur for SH and PD games.

**Figure 4:**
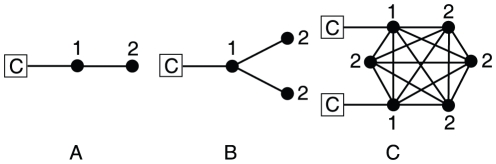
Characterization of the robustness against invasions for different topological structures. Shown are three example cases: A, a branch, B, a bifurcation, and C, a clique. Nodes indicated by a square with a C enclosed are assumed to be locked in a cooperative strategy due to their large number of connections to other cooperators (not shown).

Figure 4 presents some examples of topological traps that we find in our study. We assume that the cooperators that are shown as squares in the plots are frozen and connected to a group of cooperators such that the payoff coming from the part of the graph not shown is *k* (i.e., they have *k* cooperative neighbors, or a set of cooperators and defectors such that the net balance is *k*). To begin with, panel A shows a branch linked to cooperators. For the two dynamics we are considering here, the strategy of a given node changes (under unconditional imitation) or can change (under proportional update) only if it has a neighbor with larger payoff (i.e., players do not make mistakes). In this case, irrespective of what node 1 did at the previous round, at the next one (under unconditional imitation) or eventually (under proportional update) it will adopt the strategy C. This in turn implies that node 2, if originally a defector, can only change to cooperation if *T*<1+*S*. Note that this is also the case if node 2 is a leaf or it is connected only to defectors, as well as in many other cases. Therefore, strategy C will not be able to propagate further from 1 to 2 and beyond. The situation in Fig. 4B is similar, but in this case the condition for cooperation to propagate to nodes at position 2 is *T*<1+2*S*. Because *S*<0 we arrive at the conclusion that cooperation has even more difficulties to propagate along bifurcations, the situation becoming worse the larger the order of the bifurcation. Finally, our third example, shown in Fig.4C, illustrates the fact that propagation of strategy C can also be difficult in situations other than branches. For this particular example, nodes of type 1 will be cooperators if *k*>4*S* (*k*> (*n*−2)*S* (in case of a *n*-clique with two outwards connections), whereas nodes of type 2 will remain defectors unless *T*<1+2*S*, as in the bifurcation case above.

It is important to realize that, considering these structures as isolated ones, they will be frozen forever, no further evolution being possible with our learning rules, which do not allow for mistakes or innovative exploration of strategies. It can be shown, however, that for best response dynamics [17], [31]–[34], which is a innovative rule, similar parameter regions with coordination failures arise. On the other hand, in actual networks such as the PGP, there will be many different types of mesoscopic structures similar to those presented here. This will in turn induce a number of different thresholds of the form *T*<1+*nS*, so that different topological traps will yield to the propagation of clusters at different values of *T*, leading to the smooth transition we observe in our simulations. Our identification of such topological traps, arising from degree heterogeneity and lack of redundant paths, is further supported by the analysis presented below. As it is shown there, the PGP network has a structure consistent with our interpretation, in so far as most of its nodes are in non-redundant paths, whereas this is not the case with the URV network. In addition, we have also verified that when rewirings are restricted to nodes connected to nodes of larger degree, and only in this case, coordination failures disappear, thus pointing again at topological traps as the mechanism behind the phenomenon.

### Unveiling the structure of topological traps

The examples considered in Fig. 4 point directly to the mechanism of topological traps. In all three examples, we see that the wave of cooperation stops at nodes playing a bridging role and that are linked to other nodes with a higher degree. This immediately leads to a testable hypothesis on our two social networks: Given that coordination failures are a much more general phenomenon on the PGP network than on the URV one (where they hardly exist at all), there should be many more nodes of this class in the former than in the latter.

**Figure 5:**
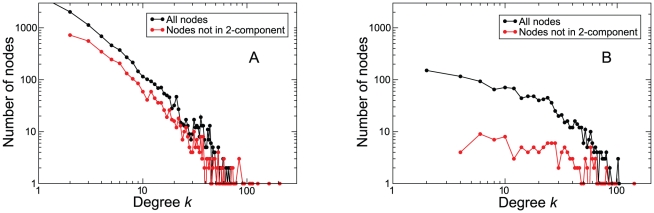
Bridging ties are the origin of coordination failures. The PGP network has many more tree-like structures than the URV network. Shown is the fraction of nodes that are not in 2-components for both empirical networks. Clearly, they are much more frequent in the PGP network (panel A, left) than in the URV (panel B, right). This is in agreement with our claim that they are responsible for the observed effect, as discussed in the main text.

First we tested the occurrence of bridging nodes sitting on unique paths for cooperation to spread. Specifically we focused on nodes that are not in 2-components. A 2-component is a subset of nodes with every node connected to 2 or more nodes of the set. Nodes that are not in 2-component are, therefore, embedded in a tree-like region, where there is no path redundancy allowing the spreading of cooperation to circumvent topological traps. Figure 5 shows that, although this kind of nodes is present in both networks, they are by far more common in PGP.

**Figure 6:**
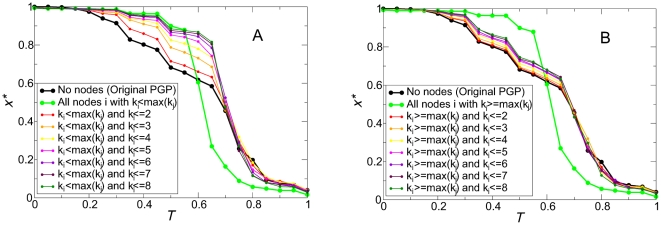
Targeted rewirings verify the degree profile of nodes involved in topological traps. Rewirings are applied separately to two complementary sets of nodes in the PGP network, namely those ones connected to at least one other node of higher degree (panel A) and all the rest, i.e. those ones with a degree equal or larger than that of any of their neighbors (panel B). In both panels thick lines correspond to the extreme cases: The original PGP network (black) and a network where all the nodes that satisfy the condition were rewired (green). Thin lines represent different intermediate cases defined by the degree of the nodes affected by the rewirings (see legends). We observe that coordination failures are strongly prevented in A but only slightly in B, supporting our claim that the former set of nodes are the ones involved in the reported phenomenon. See Methods for more details on the randomization procedures.

Second, in order to check the influence of degree heterogeneity, we carried out a new set of rewirings. We began by rewiring only those nodes that have at least one neighbor with greater degree, i.e., nodes *i* that satisfy 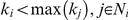 (recall that 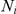 is the neighborhood of node *i*). This is done by (1) randomly finding a node which complies with this condition, (2) randomly finding any other node with degree 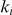, and (3) interchanging any two neighbors of them. Note that this rewiring preserves the 2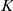-distribution, while operating only on nodes of specific degrees. With the so rewired networks, we considered the same line in the SH quadrant, focusing on how the cooperation level was affected. Panel A in Fig. 6 shows that it is certainly enough to operate on this particular kind of nodes to remove coordination failures, in a similar way as it is displayed in Fig. 2B. On the other hand, we also checked that the complementary set of nodes, i.e. those that verify 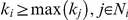, has a much slighter effect on the cooperation level. It is interesting to note that some effect cannot be avoided because any single rewiring actually affects four nodes and hence it can incidentally reconnect a low-to-high degree link.

The additional analyses described here confirm that coordination failures are caused by the two topological features that we have pinpointed above: differences in degree between neighboring nodes and lack of redundant paths, which altogether give rise to topological traps that prevent the spreading of coordination.

### Clustering and topological traps

**Figure 7:**
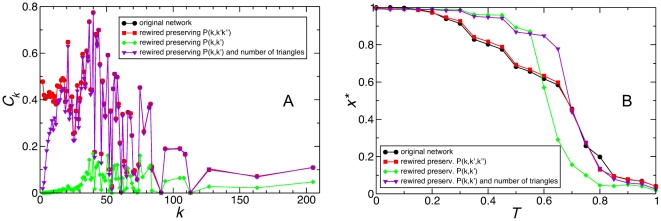
Clustering is irrelevant to topological traps. Clustering distribution per node degree *k* (panel A) and stationary density of cooperators x* as a function of game parameter *T* (panel B), for the PGP network and some rewirings presented in Fig. 2, along with a rewiring which preserves correlations of order 2 (and breaks those of order 3) while maintaining the total number of triangles in the network (see legend). Both rewirings that destroy correlations of order 3 suppress coordination failures, irrespective of the large difference in clustering. Notice that the rewiring which preserves the number of triangles only removes clustering from nodes of low degree. This removed clustering logically corresponds to the correlations of order 3 that are relevant to the reported phenomenon.

Both networks studied, PGP and URV, have some clustering [20], [21], i.e. there is some number of closed triangles of links between three nodes. In addition, triangles are very closely related to the 3*K*-distribution [35], which is precisely the order of degree correlations needed to preserve the existence of topological traps. Thus, an interesting question is to which extent clustering itself plays a role in this phenomenon. On one hand, it is virtually impossible to affect the degree distribution of clustering without changing the degree correlations of order 3, and vice versa. On the other hand, it is possible to randomize a network in a way that destroys correlations of order 3 but preserves the total number of existing triangles (see Methods). Figure 7 shows the effect of this rewiring in comparison to those used above. Panel A displays the distribution of clustering per degree *C_k_*, whereas panel B presents the the stationary state of the population. It is clear that the rewirings that preserve correlations to order 2 (and destroys those of order 3) remove the topological traps, independently of the amount of triangles in the network. Notice also that the rewiring that maintains the number of triangles reduces the clustering for low-degree nodes, something completely in agreement with the analysis presented above. Very remarkably, clustering manifests itself as a displacement of the transition between the two homogeneous population states, an effect that has been reported for other network topologies which feature clustering, namely regular lattices and small-world networks [16].

**Figure 8:**
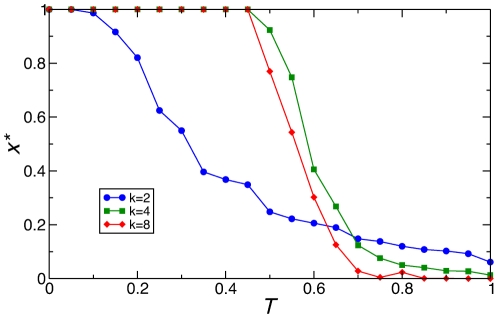
Synthetic heterogeneous networks (Barabási-Albert scale-free networks) only give rise to coordination failures when there is lack of redundant paths. Stationary density of cooperators x* as a function of game parameter *T*, for three instances of Barabási-Albert scale-free networks with degrees *k = *2, 4, and 8 (see legend). Redundant paths between nodes are only scarce for the case of *k* = 2, which is strictly a tree by construction. Notice also that clustering is very low in all three cases (mean value <0.01). Network size is *N* = 10^4^ nodes, network generation parameters are *m*_0_ = *m* = *k*/2 [54].

As an additional proof of the irrelevance of clustering for the phenomenon under discussion, Fig. 8 presents the simulation results for Barabási-Albert scale-free networks of average degrees *k* = 2, 4 and 8. This network topology features very low clustering (strictly zero for *k* = 2), while it yields strong coordination failures for *k* = 2, but none for *k* = 4 or *k* = 8. Again in agreement with our analysis, the case with *k* = 2 is the only one that, besides high heterogeneity in degree, exhibits lack of redundant paths between nodes.

## Discussion

As we have seen, social dilemmas on real social networks may exhibit largely different outcomes from those expected from model networks. In fact, this is true when social networks are compared with each other (as we have done with URV and PGP networks) but the comparison to model networks is most dramatic. A particularly striking and relevant feature arises in the case of coordination dilemmas, where certain networks, such as PGP, show coordination failures for a wide range of parameters, i.e., network regions are not able to coordinate on one of the two equilibria, leading a subset of individuals to dissatisfaction or frustration (the use of the word ‘frustration’ here is not unrelated to the manner in which it is employed in physics, for instance when speaking of antiferromagnetic Ising models or spin glasses, where many ‘spins’ cannot find a low energy configuration due to opposite sign interactions [36]). This phenomenon is observed even in the strongest case of the PD, where the range of parameters in which a high level cooperation is achieved is also affected, as the comparison between randomized and original networks shows (cf. Fig. 1).

A detailed analysis of the topology of the PGP network has allowed us to conclude that coordination failures arise from certain mesoscopic structures, more concretely from bottlenecks or topological traps that prevent the Pareto-efficient equilibrium from propagating to the whole network, effectively leading to a disruption of the information flow. The key features of these mesoscopic structures is the existence of nodes connected to nodes of higher degree, which find it difficult to induce neighbors to imitate their strategy, and the lack of redundant paths to circumvent those topological bottlenecks. The fact that the PGP network has many more of these structures than the URV network corroborates that they are responsible for the very different behavior we have found in the simulations. By the same token, the URV example shows that coordination failures are not necessarily observed on every real network, although, as we will argue below, the mechanism behind the failures seems to be quite general. In fact, this mechanism can also explain the results in [33], where small networks were exhaustively studied by numerical simulation and it was found that failures of coordination, not frequent in the sizes they studied, were related to segmentation and lack of centralization of the networks. On the other hand, the appearance of these mesoscopic structures is related to the community structure [37]–[39] of the networks. As it was discussed in [18], the PGP network consists of scale-free-like communities loosely interconnected, whereas the URV network is formed by communities which are almost complete graphs which in turn are connected almost to every other one (cf. Fig. 2 in [18], where the existence of the topological trap structures in typical PGP communities can also be appreciated). Therefore, a community analysis of a given network can provide a first indication as to whether it is going to exhibit coordination failures or not, although it must be realized that communities themselves may contain inner topological traps which could prevent them from coordinating in a unique equilibrium (as seen also in [34]).

Topological traps can indeed be a very general feature, arising in contexts related to different social issues. Thus, Castelló and coworkers [40], [41] have observed similar structures giving rise to long-lived metastable states of coexistence between languages in a population or, in other words, making consensus more difficult in opinion models. While their model cannot be exactly mapped to ours for several reasons, such as the larger number of states or the kind of strategy update dynamics, the phenomenology is quite similar and the structures we have considered here are also identified as key factors to their findings. Another instance where topological traps may play a role is the propagation of sexually transmitted diseases, which has been shown [42] to be slower when the bipartite nature of the network is taken into account; it is clear that topological traps similar to those considered here arise in a very natural manner in bipartite networks and they should certainly contribute to slowing down the transmission process. Similar phenomena can hence take place in other bipartite ecological and organizational networks [43]. In addition, mesoscopic motifs very similar to those considered here are also found in other contexts, such as communication [44], [45] and metabolic [46] networks; in this last case, considering the effect of such bottlenecks may be relevant to understand their extraordinary robustness.

In view of all these largely different examples, we envisage that topological traps will have important, non-trivial effects in almost all dynamical processes on networks, going from physical and biological to social and economical applications. This has crucial implications in two directions. First, it becomes clear that modeling actual networks with artificial models may be missing topological features as the ones we are discussing here and as a consequence lead to inaccurate results or predictions. A word of caveat is in order here: as we have seen in the case of the URV network, not all real networks exhibit topological traps. Therefore, there may be instances in which currently available model networks are adequate enough so as to account for the behavior of the system. What we claim here is that the presence of topological traps is a key feature which, when present, leads to highly nontrivial consequences. The discussion above shows that this phenomenon may arise in many real networks, and hence one should check their existence when observing anomalous behaviors on a given system. Indeed, it is particularly interesting that the reported behavior is rooted in degree correlations of order three (next-nearest neighbors), a feature whose reproduction is out of reach for most model networks. In this regard, it would be very interesting to study whether networks arising from co-evolutionary dynamics [47]–[49] exhibit this kind of structures (the persistence of defector hubs on networks arising from a PD game is a hint in this direction [50]). Second, the effect of these bottlenecks in the propagation of consensus or the diffusion of information must be kept in mind when designing networks for specific purposes, such as, e.g., innovation networks, albeit they might be useful to block diffusion processes in other contexts, most notably to prevent systemic risk in financial networks.

## Methods

### Topological null cases: Randomizations

To understand the effects of mesoscopic structure, we have compared the results on the PGP network with those obtained with several randomized versions of it. The most common randomization found in networks' literature follows the rewiring procedure proposed in [30]. The process consists of choosing randomly two nodes and exchanging one neighbor of each node (also selected randomly), which preserves the degree of each node and destroys degree-degree correlations between nodes. If the network has a well defined community structure, this process ensures its dilution. However, one can go deeper in the randomization process and preserve more quantities that just the degree of each node. In [35] the authors propose new rewiring strategies that preserve not only the original degree distribution *P*(*k*) (for simplicity 1*K* -distribution), but also the joint degree-degree distribution *P*(*k*, *k*') (2*K*-distribution) and the *P*(*k*, *k*', *k*″) (3*K* -distribution), etc. The idea is basically the same that the one followed to rewire preserving the 1*K* -distribution. When increasing the order of the correlations to be preserved, the selection of the rewired nodes is more restrictive. For example, to preserve the 2*K* -distribution, there must be at least two nodes of equal degree adjacent to the nodes in the edge pair. Then, preserving the *nK*-distribution means preserving all degree correlations up to order *n*. Generally speaking, increasing the number of correlations to be preserved shrinks significantly the possible rewirings and eventually for some *nK*-distribution the rewiring is impossible. We applied this rewiring method to the PGP network, whose limit turns out to be the 4*K*-distribution. Indeed, using a rewiring preserving the 3*K*-distribution, the resulting topology, after more than 10^4^ randomizations, is almost identical to the original one, in particular its community structure.

Here we propose a refinement of this kind of rewiring, which allows the screening of the topological characteristics in between the 2*K* -distribution and the 3*K* -distribution, in order to unravel the contribution of specific topological patterns to our results, and in the form of a partial 2*K* -rewiring. The idea is to also control the degree of the nodes involved in the trials of the edge pairs to be rewired. We can restrict the degree of the nodes that will interchange edges to any value between 2 and a greater number. For instance, we start with a rewiring that preserves the 2*K* -distribution but only nodes of degree 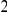 can participate in the process. This rewiring will thus break 3*K* correlations for nodes of degree 2. The process can be iterated increasing the allowed degrees, i.e. for degrees 2 and 3, for 2, 3 and 4, etc. Alternatively, one can proceed from the other end of the degree range, fixing the largest degree *d_m_* and adding progressively lower values: *d_m_* – 1, *d_m_* – 2, *d_m_* – 3, etc. Note that both strategies are complementary, but not equivalent.

The rewiring conceived to study the relevance of clustering in topological traps proceeds as follows: First, a pair of links to be shuffled is found according to the 2*K* -distribution preserving algorithm mentioned above. Then, the number of triangles of which the links are part is computed, for both cases before and after the shuffling. If the number is the same, then the rewiring is actually performed.

### Simulations

All the simulations were performed for an initial density of cooperators *x* ^0^ = 0.5. The update of strategies was done synchronously: all the individuals in the population play the game once with all their neighbors, compare payoff with them and decide the new strategy for the next time step, following one of the two rules described below. Then, they all update their strategy at once and their payoffs are set to zero before the next step. In addition, we have verified that asynchronous update leads to very similar results. The time of convergence in the simulations was *T* = 10^4^ steps. If the population did not reach full cooperation or defection, an average of the cooperator density during the last tenth of the time evolution is taken as the asymptotic cooperator density for that realization. The studied region in the *ST*-plane was sampled in steps of 0.05. For each point in the resulting 41×41 grid, which corresponds to a concrete game, 100 realizations were performed to obtain an average value of the asymptotic density of cooperators. Each realization started from a newly generated population, with strategies randomly assigned.

Our first evolution rule for strategies is the replicator rule or proportional imitation rule [51], [52]. The replicator rule is implemented as follows: let *i* = 1…*N* label the individuals in the population. Let *s_i_* be the strategy of player *i*, *π_i_* her payoff and *N_i_* her neighborhood, with *k_i_* neighbors. With the replicator rule one neighbor 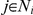 is chosen at random. The probability of player *i* adopting the strategy of player *j* is given by 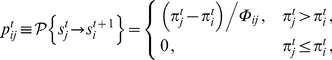
(2)with 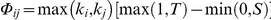 to ensure that 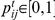.

As a second strategy update rule, we have considered another imitative rule that has received attention in previous research [11], [53], namely unconditional imitation. It makes each player choose the strategy of the neighbor with largest payoff, provided this payoff is greater than the player's one. This is a deterministic rule, in contrast to the replicator update rule, which is stochastic, and therefore it is a good reference to assess the influence of stochastic effects on our results. In addition, we have verified that results are similar with other imitative rules, such as the Moran rule [12].
